# Supplementary figures and images for: ASPPs multimerize protein phosphatase 1
Source: PLoS Genet. 2025 Oct 16;21(10):e1011731. doi: 10.1371/journal.pgen.1011731 (PMC12543280; doi:10.1371/journal.pgen.1011731)

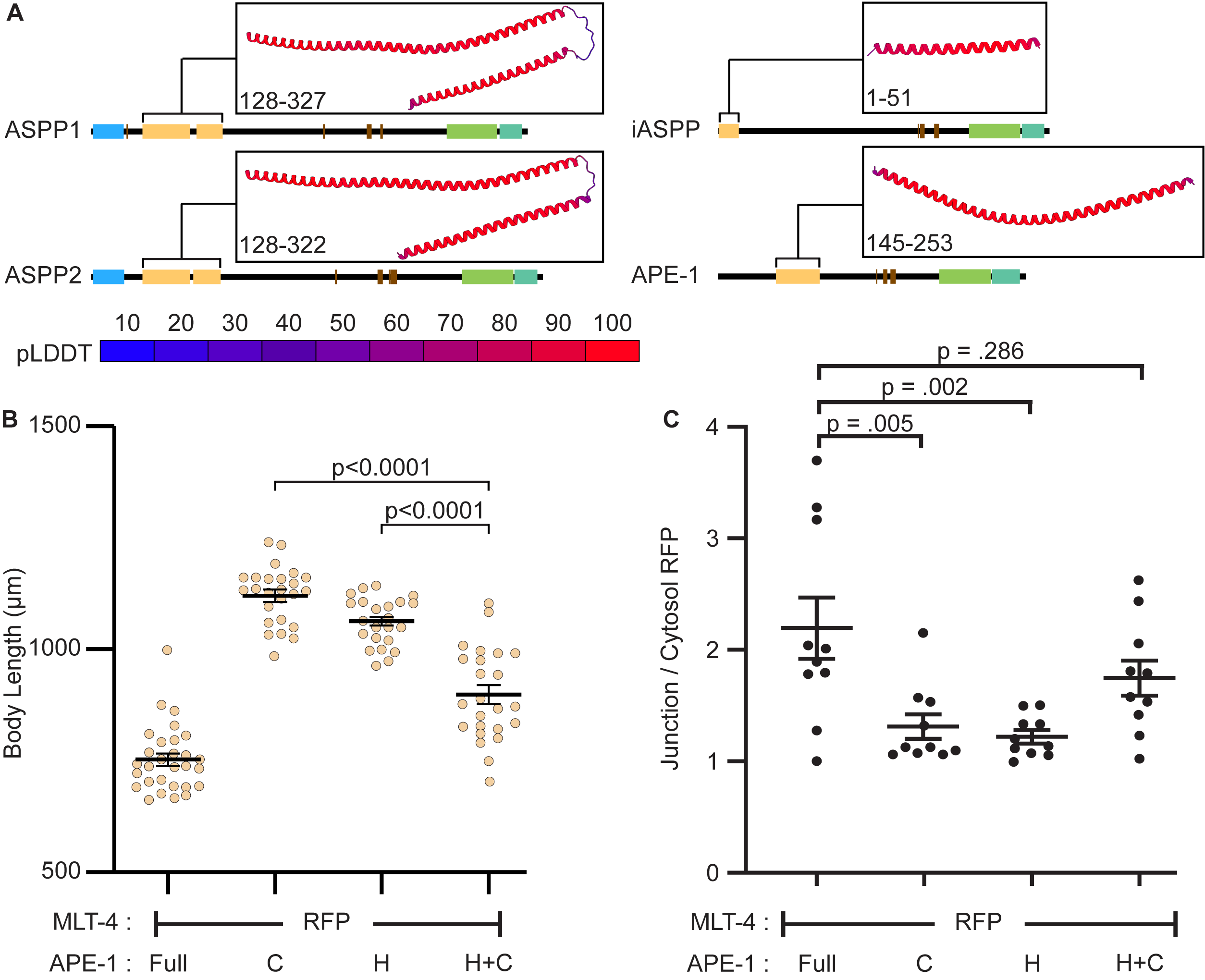

Supplement: S1 Fig — (A) Alphafold structural domain predictions mapped onto the primary sequences of ASPP1, ASPP2, and iASPP from H. sapiens as well as APE-1 from C. elegans. Predicted structures of the N-terminal alpha helices are shown in the insets above each domain map with predicted residues numbers indicated. The predicted structures are colored according to each residue’s pLDDT score provided by Alphafold. Represented in different colors are the beta-grasp domain (light blue), N-terminal alpha helix (tan), undefined alpha-helical regions (brown), ankyrin repeats (light green), and SH3 domain (teal). (B) Body length assay. Data represent mean and S.E.M. (black bars) of 23–29 biological replicates. (C) MLT-4::RFP junctional localization assay of images in Fig 1B. Data represent the mean and S.E.M. (black bars) of 10 biological replicates. p values indicated above comparison brackets. (TIF) [file pgen.1011731.s001.tif]

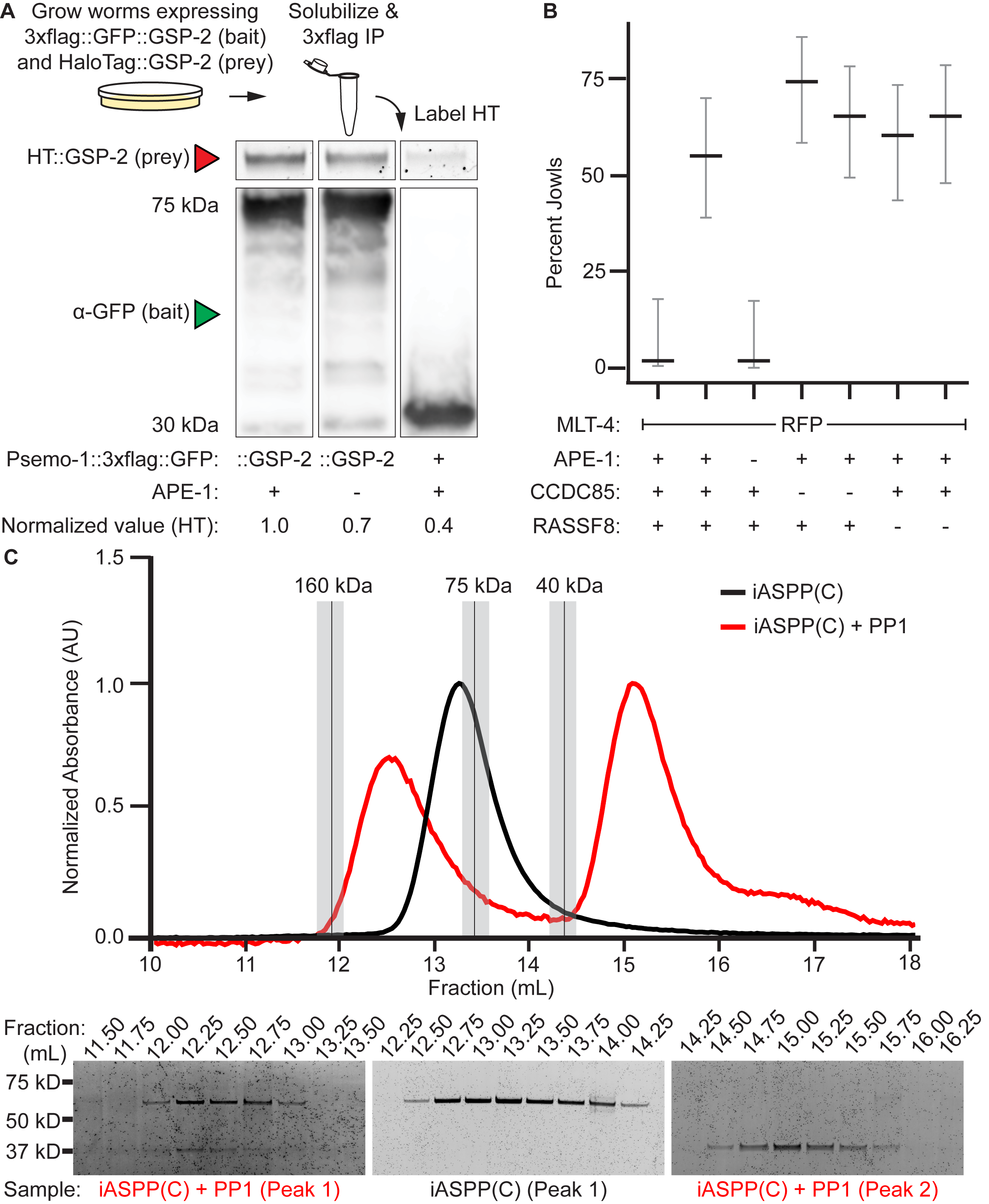

Supplement: S2 Fig — (A) 3xflag immunoprecipitations from worms expressing Psemo-1::3xflag::GFP baits and an endogenous HaloTag(HT)::GSP-2 prey. Prey is labeled with JFX646 dye (top) and bait is immunostained with anti-GFP (middle). HT intensity is normalized to the first lane (bottom). (B) Jowls assay of CCDC85 and RASSF8 knockouts (two independent alleles each). Data represent percent jowls and 95% confidence interval (black and gray bars) in adult animals (n = 40). (C) Size exclusion chromatogram of recombinantly purified iASPP(C)::HT (black line) and iASPP(C)::HT mixed with PP1 at a 1:3 molar ratio (red line; top). SYPRO Ruby-stained denaturing gels of indicated elution fractions (bottom). iASPP(C)::HT is ~ 62.7 kDA and PP1 is ~ 37.9 kDa. Peaks for molecular weight standards (described in methods) are represented as vertical black lines with the top 10% of signal shaded in light gray. (TIF) [file pgen.1011731.s002.tif]

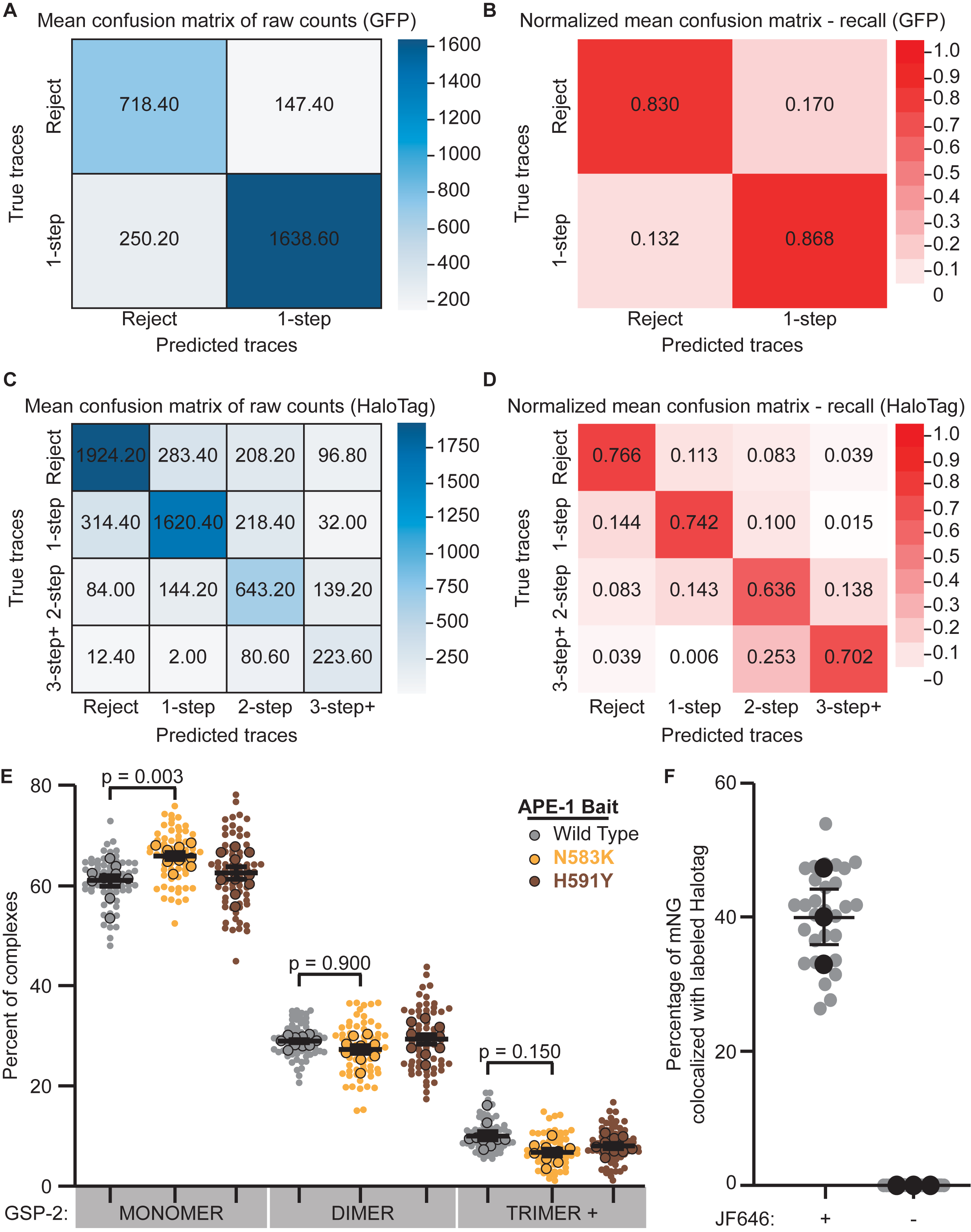

Supplement: S3 Fig — (A) CNN-Green mean confusion matrix of raw counts averaged across 5-fold cross validation during training with GFP fluorescence intensity traces. (B) Row-normalized mean confusion matrix from panel A showing recall for each class during K-fold cross validation of the CNN-Green. (C) CNN-FarRed mean confusion matrix of raw counts averaged across 5-fold cross validation during training with far-red fluorescence intensity traces. (D) Row-normalized mean confusion matrix from panel C showing recall for each class during K-fold cross validation of the CNN-FarRed. (E) Expanded view of data from Fig 4B. p values indicated above comparison brackets. Data represent mean and S.E.M. (black bars). (F) HaloTag labeling efficiency in vivo. Animals broadly expressing mNeonGreen::HaloTag under the mex-5 promoter were fed JF646 dye. Whole animal lysates were bound to functionalized coverslips via anti-mNeonGreen and imaged using TIRF microscopy. The percentage of mNeonGreen spots colocalized with far-red JF646 spots was quantified. Unlabeled animal lysates were used as a negative control. Data represent mean and S.E.M. (black bars) of three biological replicates each containing ten technical replicates. (TIF) [file pgen.1011731.s003.tif]

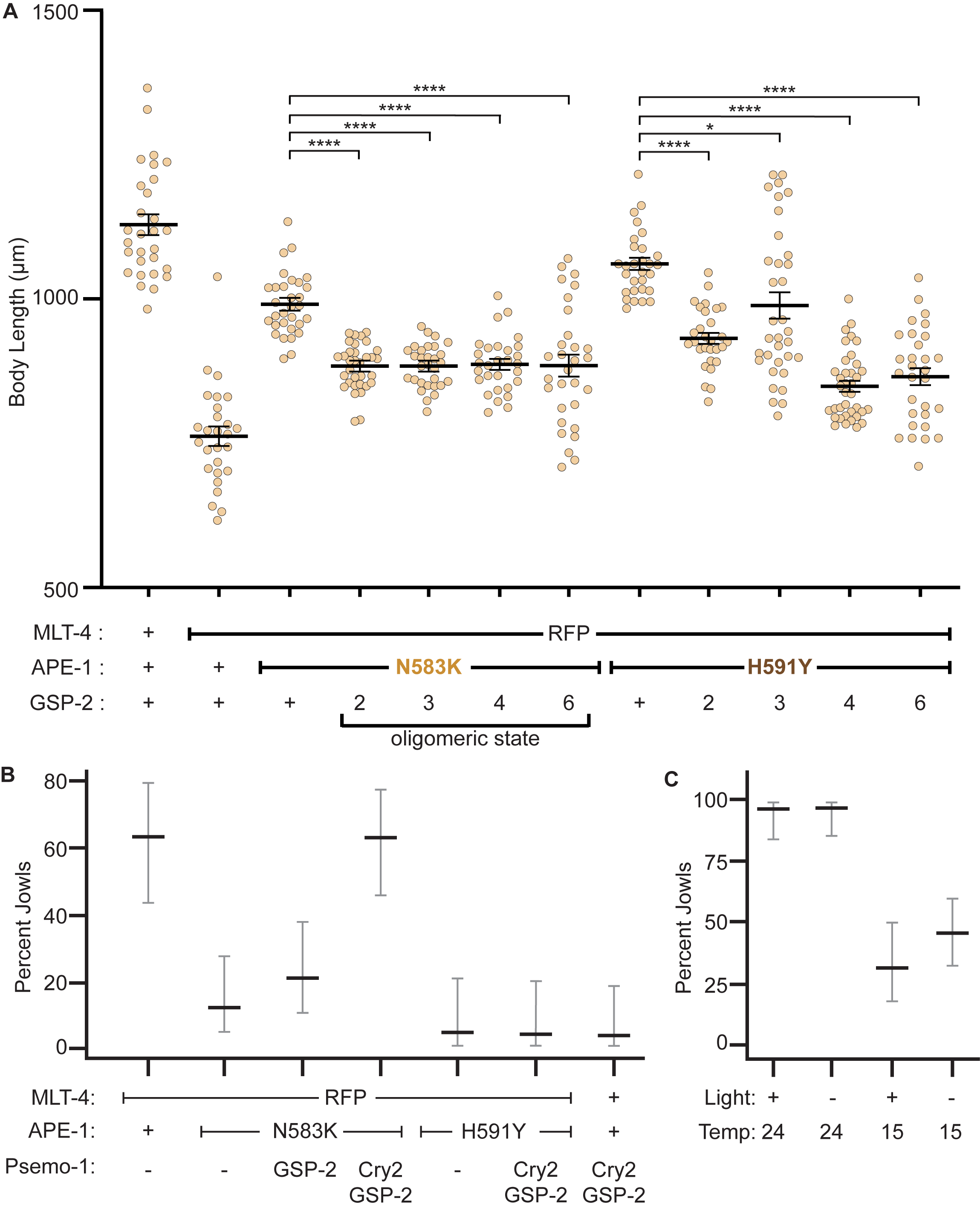

Supplement: S4 Fig — (A) Body length assay of animals with endogenous GSP-2 oligomerized via EODs. Data represent the mean and S.E.M. (black bars) of 27–36 biological replicates. **** indicates p < 0.0001. * indicates p < 0.05. (B) Jowls assay of animals expressing a skin-specific, single-copy insert of GSP-2 or Cry2olig(Cry2)::GSP-2. Data represent percent jowls and 95% confidence interval (black and gray bars) in adult animals (n = 25–35 each). (C) Jowls assay of animals expressing skin-specific Cry2::GSP-2 in MLT-4::RFP; APE-1(N583K) mutants. Animals were grown at 24°C or 15°C under light or dark conditions. Data represent percent jowls and 95% confidence interval (black and gray bars) in adult animals (n = 29–46 each). (TIF) [file pgen.1011731.s004.tif]
