# Supplementary material for: ASPPs multimerize protein phosphatase 1
Source: PLoS Genet. 2025 Oct 16;21(10):e1011731. doi: 10.1371/journal.pgen.1011731 (PMC12543280; doi:10.1371/journal.pgen.1011731)
Supplement: S3 File — (DOCX) [file pgen.1011731.s007.docx]

**Supplementary File 1**

**Strains and Alleles**

**Figure 1A & 1B**

- GUN786 *mlt-4(mew193[mlt-4::tagRFP-T::HA]) V ape-1(mew342[APE-1::GFP::3xflag]) V*
- GUN1317 *mlt-4(mew193[mlt-4::tagRFP-T::HA]) V ape-1(mew422[APE-1(520-769)::GFP::3xflag]) V*
- GUN1551 *mlt-4(mew193[mlt-4::tagRFP-T::HA]) V ape-1(mew667[APE-1(133-248)::GFP::3xflag]) V*
- GUN1604 *mlt-4(mew193[mlt-4::tagRFP-T::HA]) V ape-1(mew719[APE-1(133-248,520-769)::GFP::3xFLAG]) V*

**Figure 2A**

- GUN1661 *mewSi262[Phyp7::TIR-1::GFP::3xflag] IV; mlt-4(mew118[MLT-4(E470K)]) V*
- GUN1662 *gsp-2(mew504[3xflag::AID::GSP-2]) III; mewSi262[Phyp7::TIR-1::GFP::3xflag] IV; mlt-4(mew118[MLT-4(E470K)]) V*
- GUN1663 *gsp-2(mew504[3xflag::AID::GSP-2]) III; mewSi262[Phyp7::TIR-1::GFP::3xflag] IV; mlt-4(mew193[mlt-4::tagRFP-T::HA]) V*

**S3 Fig A**

- GUN2453 *gsp-2(mew456[Halotag::HRV::GSP-2]) III; mewSi212[Psemo-1::3xflag::GFP::GSP-2] IV*
- GUN2494 *gsp-2(mew456[Halotag::HRV::GSP-2]) III; mewSi212[Psemo-1::3xflag::GFP::GSP-2] IV; ape-1(mew1314[deletion]) V*
- GUN2454 *gsp-2(mew456[Halotag::HRV::GSP-2]) III; mewSi228[Psemo-1::3xflag::GFP] IV*

**S3 Fig B**

- N2 (Bristol)
- GUN350 *mlt-4(mew193[mlt-4::tagRFP-T::HA]) V*
- GUN658 *mlt-4(mew193[mlt-4::tagRFP-T::HA]) V ape-1(mew340[deletion]) V*
- GUN1338 *picc-1(mew594[deletion]) V mlt-4(mew193[C-terminal tagRFP-T-HA]) V*
- GUN1340 *picc-1(mew596[deletion]) V mlt-4(mew193[C-terminal tagRFP-T-HA]) V*
- GUN1341 *mlt-4(mew193[C-terminal tagRFP-T-HA]) V; K05B2.2a.2(mew597[deletion]) X*
- GUN1342 *mlt-4(mew193[C-terminal tagRFP-T-HA]) V; K05B2.2a.2(mew598[deletion]) X*
- LP539 *cpIs90[Pmex-5::mNG::HaloTag::tbb-2 3′UTR + LoxP] II*

**Figure 4A**

- GUN1573  *gsp-2(mew456[Halotag::HRV::GSP-2]) III; ape-1(mew342[C-terminal GFP 3xflag]) V*
- GUN1575 *gsp-2(mew456[Halotag::HRV::GSP-2]) III; ape-1(mew472[H591Y, C-terminal GFP 3xflag]) V*
- GUN1576 *gsp-2(mew456[Halotag::HRV::GSP-2]) III; ape-1(mew378[N583K, C-terminal GFP 3xflag]) V*

**S4 Fig**

- LP539 *cpIs90[Pmex-5::mNG::HaloTag::tbb-2 3′UTR + LoxP] II*

**Figure 5B**

- N2 (Bristol)
- GUN350 *mlt-4(mew193[mlt-4::tagRFP-T::HA]) V*
- GUN713 *mlt-4(mew193[mlt-4::tagRFP-T::HA]) V ape-1(mew345[APE-1(N583K)]) V*
- GUN2049 *gsp-2(mew1004[6HNL(2mer)::GSP-2]) III; mlt-4(mew193[mlt-4::tagRFP-T::HA]) V ape-1(mew345[APE-1(N583K)]) V*
- GUN1954 *gsp-2(mew909[3HON(3mer)::GSP-2]) III; mlt-4(mew193[mlt-4::tagRFP-T::HA]) V ape-1(mew345[APE-1(N583K)]) V*
- GUN1956 *gsp-2(mew912[YRB1(4mer)::GSP-2]) III; mlt-4(mew193[mlt-4::tagRFP-T::HA]) V ape-1(mew345[APE-1(N583K)]) V*
- GUN1958 *gsp-2(mew914[6GWK(6mer)::GSP-2]) III; mlt-4(mew193[mlt-4::tagRFP-T::HA]) V ape-1(mew345[APE-1(N583K)]) V*
- GUN711 *mlt-4(mew193[mlt-4::tagRFP-T::HA]) V ape-1(mew346[APE-1(H591Y)]) V*
- GUN2094 *gsp-2(mew1004[6HNL(2mer)::GSP-2]) III; mlt-4(mew193[mlt-4::tagRFP-T::HA]) V ape-1(mew346[APE-1(H591Y)]) V*
- GUN1953 *gsp-2(mew909[3HON(3mer)::GSP-2]) III; mlt-4(mew193[mlt-4::tagRFP-T::HA]) V ape-1(mew346[APE-1(H591Y)]) V*
- GUN1955 *gsp-2(mew912[YRB1(4mer)::GSP-2]) III; mlt-4(mew193[mlt-4::tagRFP-T::HA]) V ape-1(mew346[APE-1(H591Y)]) V*
- GUN1957 *gsp-2(mew914[6GWK(6mer)::GSP-2]) III; mlt-4(mew193[mlt-4::tagRFP-T::HA]) V ape-1(mew346[APE-1(H591Y)]) V*

**Figure 5C**

- N2 (Bristol)
- GUN350 *mlt-4(mew193[mlt-4::tagRFP-T::HA]) V*
- GUN658 *mlt-4(mew193[mlt-4::tagRFP-T::HA]) V ape-1(mew340[deletion]) V*
- GUN2360 *gsp-2(mew1234[3HON(3mer)::GSP-2]) III; mlt-4(mew193[mlt-4::tagRFP-T::HA]) V ape-1(mew340[deletion]) V*
- GUN2364 *gsp-2(mew1238[6GWK(6mer)::GSP-2]) III; mlt-4(mew193[mlt-4::tagRFP-T::HA]) V ape-1(mew340[deletion]) V*

**S5 Fig B & C**

- GUN350 *mlt-4(mew193[mlt-4::tagRFP-T::HA]) V*
- GUN713 *mlt-4(mew193[mlt-4::tagRFP-T::HA]) V ape-1(mew345[APE-1(N583K)]) V*
- GUN1762 *mewSi212[Psemo-1::3xflag::GFP::GSP-2] IV; ape-1(mew345[N583K]) V mlt-4(mew193[C-terminal tagRFP-T-HA]) V*
- GUN1740 *mewSi229[Psemo-1::CRY2olig::GFP::3xflag::GSP-2] IV; ape-1(mew345[N583K]) V mlt-4(mew193[C-terminal tagRFP-T-HA]) V*
- GUN711 *mlt-4(mew193[mlt-4::tagRFP-T::HA]) V ape-1(mew346[APE-1(H591Y)]) V*
- GUN1763 *mewSi229[Psemo-1::CRY2olig::GFP::3xflag::GSP-2] IV; ape-1(mew346[H591Y]) V mlt-4(mew193[C-terminal tagRFP-T-HA]) V*
- GUN1499 *mewSi229[Psemo-1::CRY2olig::GFP::3xflag::GSP-2] IV*

| **Allele** | **Generated by** | **Modified Locus** | **crRNA(s)** | **CRISPR repair** |
| --- | --- | --- | --- | --- |
| *mlt-4(mew118[MLT-4(E470K)]) V* | CRISPR (Beacham et al. 2022) |  |  |  |
| *mlt-4(mew193[mlt-4::tagRFP-T::HA]) V* | CRISPR (Beacham et al. 2022) |  |  |  |
| ape-1(mew340[deletion]) V | CRISPR (Beacham et al. 2022) |  |  |  |
| *ape-1(mew342[APE-1::GFP::3xflag]) V* | CRISPR (Beacham et al. 2022) |  |  |  |
| *ape-1(mew345[APE-1(N583K)]) V* | CRISPR (Beacham et al. 2022) |  |  |  |
| *ape-1(mew346[APE-1(H591Y)]) V* | CRISPR (Beacham et al. 2022) |  |  |  |
| *ape-1(mew378[APE-1(N583K)::GFP::3xflag]) V* | CRISPR | *ape-1* | rGB651 | oGB652 |
| *ape-1(mew422[APE-1(520-769)::GFP::3xflag]) V* | CRISPR (Beacham et al. 2022) |  |  |  |
| *gsp-2(mew456[Halotag::HRV::GSP-2]) III* | CRISPR (Beacham et al. 2022) |  |  |  |
| *ape-1(mew472[H591Y, C-terminal GFP 3xflag]) V* | CRISPR | *ape-1* | rGB651 | oGB655 |
| *gsp-2(mew504[3xflag::AID::GSP-2]) III* | CRISPR | *gsp-2* | rGB777 | 3xflag::AID for N-terminus of GSP-2 amplified with oGB778-oGB874 from pGB192 |
| *picc-1(mew594[deletion]) V* | CRISPR | *picc-1* | rDTW347, rDTW348 | oDTW349 |
| *picc-1(mew596[deletion]) V* | CRISPR | *picc-1* | rDTW347, rDTW348 | oDTW349 |
| *K05B2.2a.2(mew597[deletion]) X* | CRISPR | *K05B2.2a.2* | rDTW354, rDTW355 | oDTW356 |
| *K05B2.2a.2(mew598[deletion]) X* | CRISPR | *K05B2.2a.2* | rDTW354, rDTW355 | oDTW356 |
| *ape-1(mew667[APE-1(133-248)::GFP::3xflag]) V* | CRISPR | *ape-1* | rDTW282, rDTW401 | APE-1(133-248) amplified with oDTW499-oDTW500 from pDTW116 |
| *ape-1(mew719[APE-1(133-248,520-769)::GFP::3xFLAG]) V* | CRISPR | *ape-1* | rDTW581 | APE-1(133-248) amplified with oDTW582-oDTW583 from pDTW116 |
| *gsp-2(mew909[3HON(3mer)::GSP-2]) III* | CRISPR | *gsp-2* | rGB777 | 3HON amplified with oDTW801-oDTW802 from pYC26 |
| *gsp-2(mew912[YRB1(4mer)::GSP-2]) III* | CRISPR | *gsp-2* | rGB777 | Yrb1 amplified with oDTW805-oDTW806 from pYC29 |
| *gsp-2(mew914[6GWK(6mer)::GSP-2]) III* | CRISPR | *gsp-2* | rGB777 | 6GWK amplified with oDTW810-oDTW811 from pYC27 |
| *gsp-2(mew1004[6HNL(2mer)::GSP-2]) III* | CRISPR | *gsp-2* | rGB777 | 6HNL amplified with oDTW886-oDTW887 from pYC25 |
| *gsp-2(mew1234[3HON(3mer)::GSP-2]) III* | CRISPR | *gsp-2* | rGB777 | 3HON amplified with oDTW801-oDTW802 from pYC26 |
| *gsp-2(mew1238[6GWK(6mer)::GSP-2]) III* | CRISPR | *gsp-2* | rGB777 | 6GWK amplified with oDTW810-oDTW811 from pYC27 |
| *ape-1(mew1314[deletion]) V* | CRISPR | *ape-1* | rGB656, rGB657 | oGB660 |
| *mewSi262[Phyp7::TIR-1::GFP::3xflag] IV* | CRISPR |  | rGB990 | Tir-1 amplified with oGB996-oGB997 from pGB210 |
| *mewSi212[Psemo-1::3xflag::GFP::GSP-2] IV* | CRISPR |  | rGB908, rGB909 | Psemo-1 amplified with oGB911-912 from pMC08 |
| *mewSi228[Psemo-1::3xflag::GFP] IV* | CRISPR |  | rGB908, rGB909 | Psemo-1 amplified with oGB911-912 from pMC08 |
| *mewSi229[Psemo-1::CRY2olig::GFP::3xflag::GSP-2] IV* | CRISPR |  | rGB990 | Cry2olig amplified with oGB994-995 from pEB76 |
| *cpIs90[Pmex-5::mNG::HaloTag::tbb-2 3′UTR + LoxP] II* | Dickinson et al. 2017 |  |  |  |

**Plasmids**

| **Plasmid Name** | **Description** | **Generated by** | **Purpose** |
| --- | --- | --- | --- |
| pDTW116 | APE-1(66-296)::link(13)::GFP::strepII in pUC57 | Gibson cloning | Template for CRISPR repair in mew667 |
| pDTW117 | empty::link(13)::HA::link(3)::TEV::Halotag in pcDNA5frt | Beacham et al. 2022 | Figure 3C |
| pDTW123 | iASPP(602-828)::link(13)::HA::link(3)::TEV::Halotag in pcDNA5frt | Beacham et al. 2022 | Figure 2B |
| pDTW124 | iASPP(602-828, H665Y)::link(13)::HA::link(3)::TEV::Halotag in pcDNA5frt | Beacham et al. 2022 | Figure 2B |
| pDTW125 | iASPP(602-828, N657K)::link(13)::HA::link(3)::TEV::Halotag in pcDNA5frt | Beacham et al. 2022 | Figure 2B |
| pDTW131 | HA::link(3)::PPP1CA in pcDNA5frt | Gibson cloning | Figure 3B & 3C |
| pDTW154 | iASPP(602-828)::HA::TEV::Halo::6xhis in pET21b | Gibson cloning | S3 Figure C |
| pDTW231 | HA::link(3)::iASPP(602-828)::link(13)::TEV::link(5)::Halotag in pcDNA5frt | Gibson cloning | Figure 3B & 3C |
| pDTW232 | HA::link(3)::iASPP(602-828, N657K)::link(13)::TEV::link(5)::Halotag in pcDNA5frt | Gibson cloning | Figure 3B & 3C |
| pDTW233 | HA::link(3)::iASPP(602-828, H665Y)::link(13)::TEV::link(5)::Halotag in pcDNA5frt | Gibson cloning | Figure 3B & 3C |
| pDTW258 | 6xHis::HRV::PPP1CA in pET21b | Gibson cloning | S3 Figure C |
| pEB76 | Ppdi-1::Cry2olig | Gibson cloning | Template for CRISPR repair in mewSi229 |
| pGB192 | 3xflag::AID::link(3) in pUC57 | Gibson cloning | Template for CRISPR repair in mew540 |
| pGB210 | Pdpy-7::TIR-1::GFP in pUC57 | Gibson cloning | Template for CRISPR repair in mewSi262 |
| pMC08 | Psemo-1::GFP:strep::let-858 UTR | Gibson cloning | Template for CRISPR repair in mewSi212 |
| pYC25 | 3xflag::2mer(6HNL) | Chang and Dickinson 2022 | Template for CRISPR repair in mew1004 |
| pYC26 | 3xflag::3mer(3HON) | Chang and Dickinson 2022 | Template for CRISPR repair in mew909 and mew1234 |
| pYC27 | 3xflag::6mer(6GWK) | Chang and Dickinson 2022 | Template for CRISPR repair in mew914 and mew1238 |
| pYC29 | 3xflag::4mer(YrbI) | Chang and Dickinson 2022 | Template for CRISPR repair in mew912 |

**Oligonucleotides**

oDTW310 TTAACTTTAAGAAGGAGATATACATATGCCCCAAAGTATGGAAATG

oDTW349 TTTAAAGTTTTGAACTTTCTCAGGTTTCGGGTGGGAAACATTTACCAGTCACACGAAA

oDTW356 AATACATATGAATAGTCAAAATTCCCTTTCTCCTTACTTTCAAAATTTTAGCGTTCAT

oDTW457 TCCCAGACTACGCCGGCGGAAGCTCCGACAGCGAGAAGC

oDTW458 GGCGTAGTCTGGGACGTCGTATGGGTACATGATGCTAGCCAGCTTG

oDTW499 TTCCCTCCATTTTCAGATCTCAGTTCTCAACGAGATGCCTTCTGAAATGATCGCCG

oDTW500 CGCTTCCGCCGGTACCTCCACTGCCACCGCTTGTGCTGGCTAGAAGAATCTGATGTTGC

oDTW582 CAGATCTCAGTTCTCAACGAGATGCCTTCTGAAATGATCGCCGATTAC

oDTW583 TCTTCTTTCAATCGCAACTTCCATTTCGAGGTTTTGGGCTAGAAGAATCTGATGTTGCTG

oDTW801 TCTAATTGGGTTGTTTGAGCGATTTCTCGCAACAAATGAGTGGATCCTCTGGAGTCCGTC

oDTW802 GTCCAGATTAAGCTTTTCTACGTCAGATCCTCCTCTTGGAAGTGGGGTGC

oDTW805 CTAATTGGGTTGTTTGAGCGATTTCTCGCAACAAATGATGGCTCAACAGAAGCTCG

oDTW806 TTGTCCAGATTAAGCTTTTCTACGTCAGATCCTCCCTGTCCCATAGACTTCACAGAC

oDW810 CTAATTGGGTTGTTTGAGCGATTTCTCGCAACAAATGATGTCTGCTGAGAAGAAGCA

oDTW811 TTGTCCAGATTAAGCTTTTCTACGTCAGATCCTCCATCGTCAGCATCAGCAGATG

oDTW886 AATTGGGTTGTTTGAGCGATTTCTCGCAACAAATGAGTGGCAGTCATATGGCTCATCAAC

oDTW887 ATTGTCCAGATTAAGCTTTTCTACGTCAGATCCTCCGAGTGAGGCCTTGACTCCA

oDTW898 TCCGCTGCCTGGTCCCTGAAAGAGGACCTCGAGGCCGTGGTGATGGTGATGATGCA

oDTW899 TAACCGCTGAGCAATAACTAGC

oDTW900 GTTATTGCTCAGCGGTTATTTCTTGGCTTTGGCAGAATTG

oDTW901 CCAGGCAGCGGAATGTCCGACAGCGAGAAG

oDTW1113 TCCCAGACTACGCCGGCGGAAGCCCCCAAAGTATGGAAATGCG

oDTW1114 GGCGTAGTCTGGGACGTCGTATGGGTACATGATGCTAGCCAGCTTG

oEP324 ATGTATATCTCCTTCTTAAAGTTAAACAAAATTAT

oGB652 AACAGATGTCTCACAGGCCAAAGATGAAGGGATCACGGCGTTGCACAATGCGATTTGTGC

oGB655 GATGTCTCACAGGCCAATGATGAAGGGATCACGGCGTTGTACAATGCGATTTGTGCTGGA

oGB660 CCTCCATTTTCAGATCTCAGTTCTCAACGAAATGGCTCGATACATAAATCACATGTTCCG

oGB778 CTAATTGGGTTGTTTGAGCGATTTCTCGCAACAAATGAGTGACTACAAAGACCACGATGG

oGB874 GATATTGTCCAGATTAAGCTTTTCTACGTCTGTGCCACCCTTCACGAAC

oGB911 TTCCGCCGGTACCTCCACTGCCACCGCTTGTGCTCATTTTGGTTTTTGGGATTTTTGATC

oGB912 CACTTAAGGCCTTGACTAGAGGGTACCAGAGCtCAGCGTCGACCAATTTTTTTTAGCGAG

oGB994 TGGAGACGCTTCCGCCGGTACCTCCACTGCCACCGCTTGTGCTGGCGGCTCCGATCATGA

oGB995 ATCAAAAATCCCAAAAACCAAAATGAAGATGGACAAGAAGACCATCGTC

oGB996 ATCAAAAATCCCAAAAACCAAAATGCAGAAAAGGATTGCATTGTCGTTTC

oGB997 GGAGACGCTTCCGCCGGTACCTCCACTGCCACCGCTTGTGCTGAGTCCGTTGGTGGTGAT

**CRISPR guides**

rDTW282 GATCTCAGTTCTCAACGAGA

rDTW347 AACTTTCTCAGGTTTCGAAG

rDTW348 AACTAATATCCACTCTAGGT

rDTW354 AGCTCCATTTCTGCAAGAAA

rDTW355 ATTTTGAAAGTAAGGAGCTC

rDTW401 AACAATGTCGAAAGTACTAG

rDTW581 CGAGATGCAGAATCTTGAAA

rGB651 GGCCAATGATGAAGGGATTA

rGB656 GATCTCAGTTCTCAACGAGA

rGB657 CGAACAACAATGTCGAATAA

rGB777 GAGCGATTTCTCGCAACAAA

rGB908 CGTGGAATGAGACTGTGCTC

rGB909 TCCAGATAAATGAGTACTAG

rGB990 ACCAAAATGAGCACAAGCGG
