# Supplementary material for: ASPPs multimerize protein phosphatase 1
Source: PLoS Genet. 2025 Oct 16;21(10):e1011731. doi: 10.1371/journal.pgen.1011731 (PMC12543280; doi:10.1371/journal.pgen.1011731)
Supplement: Attachment — Submitted filename: Response to Reviewers.pdf [file pgen.1011731.s009.pdf]

Dear Dr. Jeremy Nance,

Thank you for overseeing the review of our manuscript and providing us with clear guidance on how to proceed with our revision process.

We also thank the reviewers for their time and insightful comments that helped us to improve and clarify our manuscript.

With the suggestions from the reviewers, we have generated a substantial amount of new data included across four supplemental figures. In particular, to exclude the possibility that CCDC85 and RASSF8 cooperate with the ASPPs in oligomerizing PP1, we generated multiple CRISPR deletion alleles to assess in our jowls assay. Additionally, we were able to quantify our labeling efficiency of HaloTag in our TIRF microscopy, which revealed that we are likely underestimating the amount of phosphatase in our single molecule experiments. Finally, we attempted to reconstitute the superstoichiometric ASPP:PP1 complex *in vitro* using proteins purified from bacteria. However, size exclusion chromatography suggests a 1:1 complex, as has previously been shown in crystal structures. This may be due to the fact that PP1 purified this way contains manganese ions in the active site leading to aberrant function, whereas native phosphatase is hypothesized to contain iron and magnesium.

Below, we have addressed all of your concerns point-by-point. We have included a copy of the manuscript with all changes highlighted in yellow. We hope you agree that the manuscript has been significantly improved by this revision process.

Reviewers' comments:

Reviewer's Responses to Questions

Reviewer #1: The manuscript by Wei and colleagues is a well-focused study demonstrating that ASPP proteins likely act by oligomerizing PP1 (Ser/Thr phosphatase) enzymes. In addition, The C. elegans N-terminus of APE-1 is required for its localization to apical junctions located between hyp7 and the seam cell of the worm epidermis. A major strength of the study are the multiple complementary methods used to show that ASPP proteins promote PP1 oligomerization through a domain in the C-terminus and that this impacts the function of APE-1/ASPP–GSP-2/PP1 based on functional readouts in the worm. Moreover, induced oligomerization of GSP-2 bypasses defects in the C-terminus of APE-1, demonstrating that oligomerization is really a crucial component of the function of APE-1 in addition to localizing PP1 to apical junctions. The paper is concise and well written with carefully drawn conclusions and should be of considerable interest to several fields. I have no requested experiments, and my relatively few comments and questions may be viewed as suggestions.

1) Fig 1. The signal in the representative image for MLT-4::RFP–H appeared a good bit dimmer than the other images. Was this a trend and, if so, does it suggest that expression of APE-1–H somehow affects the ability of MLT-4 to associate with these junctions?

We thank the reviewer for noticing the trend of MLT-4::RFP appearing dimmer in our APE-1 mutants. We agree that MLT-4::RFP does have a decreased localization to the epithelial junctions in the cases of APE-1 being mutated. To address this question, we quantified the ratio of MLT-4::RFP at the epithelial junctions compared to that in the cytosol by duplicating the

junctional ROI we had previously drawn for APE-1 quantification and moving it to a nearby cytosolic region. We compared the mean intensities across these two ROI's in the red channel and found that MLT-4 is significantly reduced at the junctions when the APE-1 C-terminus or Helix is deleted (S1 Fig C).

These data suggest that APE-1 may have some role in facilitating the localization of the Inversin complex to epithelial junctions – the exact details of which remains unknown. This observation offers an opportunity for an interesting future study.

Results; Line 126 – "We also noted that MLT-4::RFP intensity was reduced in these APE-1 truncations (S1 Fig C), suggesting APE-1 activity may also facilitate MLT-4 localization."

2) Fig S1. I was curious what the pLDDT view of the helices in S1 Fig might look like. My understanding is that AF likes to create helices that are very low confidence even when regions are potentially unstructured. So, I was curious about the confidence of those predictions and if there is any experimental support for helices, such as crystal structures, which were mentioned in the Discussion.

We agree that the pLDDT values for the Helices are key information to include. We have overlaid heat maps indicating pLDDT values on the predicted structures and included a scale bar (S1 Fig A). Most regions of the Helices were predicted with high confidence (pLDDT > 80) with the exception of a small linker region observed in the ASPP1 and ASPP2 Helices.

The crystal structures mentioned in the discussion are all of the C-terminal ankyrin repeats and SH3 domain. To our knowledge, there are no structural data to support the AlphaFold predictions of Helices in the ASPP N-termini.

3) Fig 2. Consider adding some basic information about the nature of the two iASPP-C mutations to the figure legend, as these are not currently mentioned.

We agree with the reviewer, Fig 2 appears before the description of these missense mutations in the manuscript. We adjusted the caption of Fig 2 to include the following sentence

Fig 2 caption; line 995 – "Missense mutations (brown and orange) identified from a forward genetic screen, which each inhibit APE-1 in *C. elegans*, were also included in the iASPP bait."

4) Fig 3. In 3C it isn't immediately clear what the label Halotag indicates since all the constructs should have the tag. Perhaps list it as "control" and provide an estimated molecular weight, as this is not stated in the methods.

We have included a sentence to clarify the sample description in the figure caption.

Fig 3 caption; line 1003 – "Fluorescent size exclusion chromatography of HaloTagged (HT) proteins labeled with JFX549 in HEK293T cell lysates."

We have also included a sentence in the figure caption to clarify what the HaloTag control is and its molecular weight.

Fig 3 caption; line 1007 – “The HaloTag negative control protein (gray line) is approximately 37.2 kDa.”

5) Fig 4. Although the data in 4B clearly indicate that the proportion of oligomeric (2 or more) complexes decreases with the N583K mutation, it does so by less than one might expect given some of the other data. In addition, the confusion matrices in S4 suggest a decent ability to distinguish between 2 and 3 steps. So, it was unclear why these weren’t compared among the genotypes. For example, did H591Y show any distinction between 2 and 3 versus WT? Even if differences were not observed, it could still be informative to show that data (as a sup) and to try and account for why the different techniques, while agreeing generally, didn’t correlate especially well. Admittedly, this was touched upon in the Discussion, but a clear hypothesis for the discrepancies wasn’t forwarded. (Perhaps there is none.)

We thank the reviewer for this helpful suggestion. We further segregated the data to differentiate between complexes containing dimeric and trimeric GSP-2 (S4 Fig E). Since the higher order classes are detected less frequently, there is no statistical difference between our mutants and WT complexes. We added a statement to the results to address this point.

Results; line 257 – “To evaluate if the missense mutations stabilize a specific GSP-2 oligomeric state, we segregated the data to quantify the distribution of GSP-2 monomers, dimers, and trimers-or-higher (S4 Fig E). By this analysis, there is no statistically significant difference between the wild type and the missense mutants in the higher order complexes.”

We agree that the differences between WT and the mutants are less than we expected. Thanks to the suggestion from another reviewer, we evaluated the labeling efficiency of the HaloTag in our TIRF microscopy (S4 Fig F). Our data suggest that our labeling efficiency is approximately 40%. Therefore, our experiments are likely underestimating the abundance of GSP-2 in ASPP:PP1 complexes, especially in higher order oligomers of GSP-2. This decreased probability of detecting GSP-2 oligomers could explain why the TIRF microscopy results are less robust than our other assays. We added a statement explaining these data and this hypothesis in the results.

Results; line 261 – “Our single-molecule analysis is highly dependent on efficiently labeling the HaloTag::GSP-2 with fluorescent dye. Since our labeling efficiency is approximately 40% (S4 Fig F), we are likely underestimating the number of phosphatase molecules, and may fail to detect higher order ASPP:PP1 complexes.”

We also included a statement explaining apparent discrepancies between our single-molecule TIRF microscopy and other *in vitro* assays.

Discussion; line 353 – “Finally, because our labeling efficiency is only 40% (S4 Fig F), our single-molecule TIRF microscopy analysis is likely underestimating the number of GSP-2 in many ASPP:PP1 complexes.”

6) Discussion. Connected point #5, might the defect in H591Y have more to do with the orientation of the oligomerized PP1 subunits, such that while forming oligomers they are still inactive? By chance, were any other kinds of EODs tested that failed to work? Also, what was the origin of the EOD used in this study and why was it chosen?

Excellent idea. We had initially chosen the EODs based on their extensive characterization by the lab of Dr. Dan Dickinson (Chang and Dickinson 2022). They selected for proteins that when fused to mNeonGreen in *C. elegans*, produced dimeric, trimeric, tetrameric, or hexameric

step-wise photobleaching in their single-molecule TIRF microscopy experiments. As an alternative oligomerization method, we fused Cry2olig to GSP-2. Cry2olig is a modified Cryptochrome 2 from *Arabidopsis thaliana* and has been shown to homo-oligomerize to variable sizes when stimulated with blue light (Taslimi et al. 2014). We show that fusing Cry2olig to GSP-2 restored jowls to animals with the N583K missense mutation in APE-1 (S5 Fig B). However, the Cry2olig did not restore jowls to animals with the H591Y missense mutation. These data are consistent with your model whereby the Cry2olig drives a GSP-2 cluster that is incompatible with the H591Y mutant. A note regarding the Cry2olig – we do not observe light sensitivity, but rather temperature sensitivity in our system (S5 Fig C).

We included a statement regarding these data in the results.

Results; line 284 – “We also tested an alternative EOD, a modified *Arabidopsis* thaliana Cryptochrome 2 (Cry2olig) that drives protein clustering in response to light [46]. Fusing Cry2olig to GSP-2 restored jowls in the N583K, but not in the H591Y missense mutants (S5 Fig B), suggesting that these two mutations may inactive the ASPP:PP1 complex via slightly distinct mechanisms. One caveat is that the Cry2olig did not exhibit light sensitivity, but rather temperature sensitivity – at room temperature animals exhibited jowls both in the light and dark, whereas the jowls were suppressed at 15°C (S5 Fig C).”

We also included a statement regarding the hypothesis that N583K and H591Y may act via different mechanisms, namely the orientation of GSP-2 may be important.

Discussion; line 365 – “However, stimulating oligomerization of GSP-2 with Cry2olig bypassed the N583K missense mutant, but not the H591Y (S5 Fig B). These data suggest there may be an additional level of regulation, perhaps a specific orientation of phosphatase subunits, which the H591Y mutant cannot achieve.”

As for all my reviews this is signed by David Fay.

Reviewer #2: Protein phosphorylation is a critical post-translational modification that influences cell signaling and behavior. Regulation of the protein kinases that catalyze phosphorylation has been extensively studied, but regulation of phosphatases, which reverse this modification, is comparatively underexplored. Here, Wei and colleagues have used genetic and biochemical experiments to study regulation of protein phosphatase 1 (PP1) by ASPP proteins and their C. elegans homolog, APE-1. Their data show that ASPP recruits PP1 into clusters, and this clustering is necessary and sufficient for normal PP1 activity in C. elegans.

This a really nice, straightforward paper that integrates complementary approaches to reach an interesting result. Indeed, the manuscript was so easy to read that while reading, it was tempting to forget what tremendous amount of work must have gone into combining engineered loss- and gain-of-function experiments, structure-function approaches, and both bulk and single-molecule biochemistry into a such a cohesive story. Well done. I have no major concerns about the manuscript and only a few minor points for the authors to consider:

- It would be nice if the authors could be a bit more precise about the molecular weights they expect in their fSEC experiments. They write “A 1:1 ratio [of iASPP to PP1] would be ~100 kDa.” According to Uniprot, iASPP is 89 kD and PP1 is 36 kD, so a 1:1 ratio would be 125 kD. Also, if possible, it would be good to provide a control for how unbound, monomeric iASPP runs on their column – perhaps by doing the separation under denaturing conditions or by starting with cells depleted of PP1.

We apologize for failing to clarify the molecular weights of each protein used in Fig 3C. Note that our construct of HA::iASPP(602-828)::TEVsite::HaloTag expressed in the HEK cells only consists of the C-terminal ankyrin repeats and SH3 domain. Together with the tags, our iASPP C-terminal constructs have a predicted molecular weight of 62.1 kDa. Our construct of HA::PP1 used in this experiment is 38.8 kDa. We added a statement to clarify the molecular weights in the caption.

Fig 3C caption; line 1006 – "PP1 is 38.8 kDa and iASPP(C)::HT is 62.1 kDa."

We agree that a control showing how monomeric iASPP C-terminus runs on its own would be good to include. While we did not repeat the fSEC under denaturing conditions or in cells depleted of PP1, as part of a response to another reviewer we purified a recombinant iASPP C-terminal construct (iASPP(602-828)::HA::TEVsite::HaloTag::6xHis) from bacteria and ran it on size exclusion chromatography (S3 Fig C, top). The iASPP C-terminal construct elutes from the column just prior to the conalbumin control (75 kDa), which suggests the iASPP C-terminal construct exhibits a slightly larger molecular weight than is predicted (62.7 kDa).

- The fSEC and SiMPull measurements show the same trend, which is nice, but I was surprised not to see more larger clusters in the SiMPull experiments. How efficient is HaloTag labeling on worm plates, and could under-counting of HaloTags due to incomplete labeling explain why there aren't more larger clusters? If the authors don't have an estimate of labeling efficiency under their conditions, they can simply acknowledge the possibility that labeling is incomplete as a possible caveat.

We thank the reviewer for the excellent suggestion of determining our labeling efficiency in our SiMPull experiments. We performed HaloTag labeling on the *C. elegans* strain LP539 (graciously provided by Dr. Dan Dickinson), which expresses mNeonGreen::HaloTag under the *mex-5* promoter. Our functionalized coverslips pulled on the mNeonGreen with a biotinylated anti-mNeonGreen. We imaged these coverslips as described in our other TIRF microscopy experiments and quantified the percent colocalization of mNeonGreen spots with the far-red labelled HaloTag spots. Our labeling efficiency averaged at approximately 40% across three biological replicates, each containing ten technical replicates (S4 Fig F). This lower labeling efficiency suggests that we are likely underestimating the presence of GSP-2 in our SiMPull experiments, as the reviewer proposes.

We added a statement explaining these data in the results.

Results; line 261 – "Our single-molecule analysis is highly dependent on efficiently labeling the HaloTag::GSP-2 with fluorescent dye. Since our labeling efficiency is approximately 40% (S4 Fig F), we are likely underestimating the number of phosphatase molecules, and may fail to detect higher order ASPP:PP1 complexes."

We also included a statement explaining apparent discrepancies between our single-molecule TIRF microscopy and other *in vitro* assays.

Discussion; line 353 – "Finally, because our labeling efficiency is only 40% (S4 Fig F), our single-molecule TIRF microscopy analysis is likely underestimating the number of GSP-2 in many ASPP:PP1 complexes."

- Along the same lines, the authors trained their classifier to distinguish dimers from multimers, but then didn't report the results of that classification with respect to GSP-2. Were complexes

with 3+ GSP-2 subunits ever observed, and if so, how often? It would be good to report this (perhaps in the supplement).

We thank the reviewer for this suggestion that was proposed by all reviewers. To address this question, we further segregated the data to differentiate between complexes containing dimeric and trimeric-or-higher GSP-2 (S4 Fig E). We did observe trimeric and higher complexes in all three samples (~10%). However, since the higher order classes are detected less frequently, there was no statistical difference between our mutants and WT complexes. We added a statement to the results to address this point.

Results; line 257 – “To evaluate if the missense mutations stabilize a specific GSP-2 oligomeric state, we segregated the data to quantify the distribution of GSP-2 monomers, dimers, and trimers-or-higher (S4 Fig E). By this analysis, there is no statistically significant difference between the wild type and the missense mutants in the higher order complexes.”

Reviewer #3: In the present manuscript, Wei et al use C. elegans genetics and biophysical techniques to study the role of the scaffold protein APE-1 (ASPP family member iASPP in mammals) in regulating the activity and localisation of the PP1 catalytic subunit. They show that APE-1 associates with cell junctions via its N-terminal helical region and confirm previous findings that ASPP proteins associate with PP1 through their C-terminus. Unexpectedly, the authors uncover evidence that APE-1 nucleates PP1 oligomerisation. They identify loss-of-function mutations in the ASE-1 ankyrin repeats that affect its ability to associate with super-stoichiometric amounts of PP1 using co-immunoprecipitations, gel filtration and a TIRF assay. The data are generally solid and well presented. The ability of a regulatory subunit to promote PP1 oligomerisation is a new observation that adds to our understanding of how phosphatase activity is targeted to the correct subcellular localisation to ensure appropriate substrate dephosphorylation. The study should therefore be of broad interest, however the points below should be addressed in order to strengthen the authors’ conclusion before the manuscript can be accepted in PLoS Genetics.

1) Looking at the published structures of iASPP/PP1 (ref 45) and ASPP2/PP1 (ref 24), it is not clear how several PP1 subunits could associate simultaneously with ASE-1. Could the authors model this possible association using AlphaFold to propose some possible scenarios that might allow this to happen?

We agree that AlphaFold and other predictive software could improve our understanding of the ASPP:PP1 complex. To address this point, we submitted 721 jobs to the AlphaFold 3 server testing many ratios of iASPP or APE-1 paired with PP1 or GSP-2, respectively. 1:1 models receive an interface predicted template modeling (iPTM) score of 0.81 or greater, which is considered a confident prediction. All other models receive an iPTM score of 0.51 or less, which is considered a failed prediction. These models constitute ~440 GB of data and we are happy to include them as a supplement if the reviewer feels they would contribute positively to the manuscript. However, we have not included these data at this point as we assume these models will become obsolete within a year given the rapid progress of structural prediction algorithms.

2) Along the same lines, although the authors do a good job of using orthogonal approaches to validate their model of PP1 oligomerisation, none of the experimental setups exclude that for instance other proteins associated with the ASPP/PP1 complex (e.g. CCDC85 and RASSF family members in the case of ASPP2/Drosophila ASPP) could participate in PP1 oligomerisation. For example, the gel filtration experiments are consistent with multiple PP1

subunit being present in the slower-migrating complexes, but other proteins could also cause (part of) this shift and be sensitive to the N583K mutation.

We agree that we cannot exclude the possibility of other proteins participating in the ASPP:PP1 complex. To rule out known ASPP interactors, we used CRISPR to generate CCDC85 and RASSF8 knockout animals and observed that two independent alleles for each failed to suppress the jowls phenotype (S3 Fig B). We believe this is strong genetic evidence that CCDC85 and RASSF8 are not required for APE-1 function in our *C. elegans* system.

To address this point, we included a statement in the results.

Results; line 216 - “Perhaps other proteins cooperate with the ASPPs to promote phosphatase oligomerization. For instance, CCDC85 and RASSF8 are ASPP interactors hypothesized to regulate cellular junctions via PP1 in Drosophila melanogaster [24,36]. Given that two independent deletion alleles for each CCDC85 and RASSF8 failed to suppress the jowls phenotype (S3 Fig B), it appears that neither are involved in ASPP oligomerization of PP1 in our system.”

I would therefore suggest the following experiments:
- Perform the TIRF assay in Figure 4 using the ASE-1 C-terminal construct. This would reduce the likelihood that other ASE-1 partner are participating in oligomerisation.

We apologize that it was unclear that both our fSEC and quantitative pulldowns of HEK samples were performed with an iASPP C-terminal construct that only contained the ankyrin repeats and SH3 domain. We clarified this point in the Figure 3 caption for our gel filtration experiment.

Fig 3 caption; line 1006 - “PP1 is 38.8 kDa and iASPP(C)::HT is 62.1 kDa. The HaloTag negative control protein (gray line) is approximately 37.2 kDa.”

Since both our fSEC and quantitative pulldowns show the ASPP N-terminus is dispensable for ASPP-dependent oligomerization, we did not genome engineer additional *C. elegans* strains to similarly test the requirement of the N-terminus in our TIRF assay.

- Perform the TIRF assay in Figure 4 by tethering a Flag-GFP-tagged PP1 and imaging HALO-tagged PP1. This would test the ability of PP1 itself to oligomerise.

We thank the reviewer for this suggestion. While we did not image both phosphatase constructs on our TIRF microscope, we immunoprecipitated a single-copy, skin-specific, 3xflag::GFP::GSP-2 and did observe that the endogenous HaloTag::GSP-2 co-precipitated more so than in an APE-1 knockout and in the 3xflag::GFP negative control (S3 Fig A). This result is consistent with the possibility PP1 itself can oligomerize, which offers an interesting opportunity for future studies. In addition to S3 Fig A and its caption, we included two statements in the results.

Results; line 213 - “We found that a single-copy, skin-specific, 3xflag::GFP::GSP-2 bait coprecipitated with endogenous HaloTag::GSP-2 (S3 Fig A). Knockout of APE-1 reduced the amount of HaloTag::GSP-2 coprecipitation, but not to the level of the 3xflag::GFP negative control bait.”

Results; line 221 - “It is also possible that the residual oligomerized phosphatase in our precipitations may represent other, ASPP-independent, complexes.”

- In an ideal world, the authors would perform biophysical assays/gel filtration and/or GST pulldowns on bacterially expressed and purified ASE-1 C-ter and PP1 to prove the oligomerisation model in a "clean" system, though I realise they may not have these approaches established in their lab.

We agree that reconstitution of an oligomeric ASPP:PP1 complex *in vitro* would be the best evidence for our model. We developed the methodology to express and purify the iASPP C-terminus and PP1 from bacteria. However, we were unable to reconstitute the superstoichiometric complex by mixing these components and evaluating the size of the resulting complex by size exclusion chromatography (S3 Fig C). At this point, our leading hypothesis as to why our *in vitro* reconstituted complex exhibited a 1:1 stoichiometry (as the crystal structures show) is that PP1 purified from bacteria is known to contain the wrong metal ions in its active site, leading to aberrant activity. However, it is also possible we are lacking some additional factor, such as another protein, required for PP1 oligomerization *in vitro*. We added the following statements.

Results; line 223 - "If the ASPP-dependent oligomerization of PP1 requires no other eukaryotic proteins, then it may be possible to reconstitute the complex using components purified from bacteria. We mixed recombinantly purified iASPP C-terminus and PP1 at a 1:3 molar ratio, respectively (S3 Fig C). Using size exclusion chromatography, we observed that the ASPP:PP1 complex eluted at a size consistent with a 1:1 stoichiometry, which is reminiscent of the iASPP(602-828, N657K) complex in the fSEC data (Fig 3C). The lack of evidence of oligomerized PP1 using recombinant proteins suggests that we are missing some key factor for oligomerization. It has been established that PP1 purified from bacteria has non-native metal ions in its active site and exhibits inappropriate activity [44], which may account for our inability to reconstitute the superstoichiometric complex. However, we cannot exclude the possibility that PP1 oligomerization depends on additional proteins working with the ASPPs."

I should say that if it does prove that for example other proteins are involved in forming PP1 oligomers, this would not preclude publication. These experiments are suggested to help the authors refine their model.

We greatly appreciate the reviewer for pointing out that failure to exclude the possibility of other participating proteins does not preclude publication of our study.

3) Figure 4B: for the readers to better appreciate the difference between the mutant and wt APE-1, it would be good to provide a supplementary graph showing the relative distribution of the different oligomeric forms (ASE-1+1/2/3+ PP1) in mutants vs wt rather than just the percentage of complexes with oligomers as shown in the figure.

We thank the reviewer for this suggestion, which was consistently shared by all reviewers. We further segregated the data to differentiate between the different oligomeric forms of phosphatase (S4 Fig E). Since the higher order classes are detected less frequently, there is no statistical difference between our mutants and WT complexes. We added a statement to the results to address this point.

Results; line 257 – "To evaluate if the missense mutations stabilize a specific GSP-2 oligomeric state, we segregated the data to quantify the distribution of GSP-2 monomers, dimers, and

trimers-or-higher (S4 Fig E). By this analysis, there is no statistically significant difference between the wild type and the missense mutants in the higher order complexes."

During the review process, we found that our labeling efficiency of HaloTag was ~40% (S4 Fig F). These data suggest we are underestimating the amount of phosphatase present in our TIRF microscopy experiments.

Results; line 261 – "Our single-molecule analysis is highly dependent on efficiently labeling the HaloTag::GSP-2 with fluorescent dye. Since our labeling efficiency is approximately 40% (S4 Fig F), we are likely underestimating the number of phosphatase molecules, and may fail to detect higher order ASPP:PP1 complexes."
